# A Fresh View on Limestone Calcined Clay Cement (LC^3^) Pastes

**DOI:** 10.3390/ma14113037

**Published:** 2021-06-03

**Authors:** Hassan Ez-zaki, Joseph Mwiti Marangu, Maurizio Bellotto, Maria Chiara Dalconi, Gilberto Artioli, Luca Valentini

**Affiliations:** 1 CIRCe, Department of Geosciences, University of Padova, Via G. Gradenigo 6, 35131 Padova, Italy; mariachiara.dalconi@unipd.it (M.C.D.); gilberto.artioli@unipd.it (G.A.); luca.valentini@unipd.it (L.V.); 2 Department of Physical Sciences, Meru University of Science & Technology, Meru P.O. Box 972-60200, Kenya; jmarangu@must.ac.ke; 3 Opigeo S.r.l., Via Monte Cengio, 28/1, 35138 Padova, Italy; maurizio.bellotto@opigeo.eu

**Keywords:** limestone calcined clay cement, LC^3^, yield stress, plastic viscosity, rheology, plastic shrinkage

## Abstract

In this work, the factors controlling the fresh state properties of limestone calcined clay cement (LC^3^) are assessed and compared to Portland and binary cements, extending the scope of previous research by combining rheological measurements with setting time determination and the evaluation of plastic shrinkage by a novel method. Yield stress and elastic modulus are considered indicators for the structural build-up/breakdown process when stress is applied to the system. On the other hand, plastic shrinkage occurs from the mixing to the setting of fresh paste and plays an important role in governing microstructural changes due to settlement and evaporation. Evaluation of the rheological properties with time was appropriate to give an overview of the influence and behavior of different added materials. The elastic modulus of all binders (clinker, LC^3^, clinker–limestone, and clinker–calcined clay) was increased from mixing to 60 min of curing as follows: 5.27 × 10^3^ to 9.50 × 10^5^ Pa, 5.94 × 10^3^ to 9.87 × 10^5^ Pa, 6.89 × 10^3^ to 5.62 × 10^5^ Pa and 7.85 × 10^3^ to 1.27 × 10^6^ Pa, respectively. Moreover, during the first three hours of curing, LC^3^ exhibited a reduction of plastic shrinkage by more than a factor of 2 compared to clinker cement. The use of calcined clay with clinker increases the elastic modulus of the system due to the flocculation effect and increased water absorption, while a dilution effect is contributed due to deflocculation and a free-water increase in the system when a high fraction of limestone is present in the binary cement. The combination of limestone and calcined clay with clinker can induce additional chemical reactions, which control the early age properties, such as plastic shrinkage. The obtained results can contribute to optimizing the fresh state properties of ternary blends of OPC, calcined clay, and limestone through a knowledge-based approach.

## 1. Introduction

One of the reasons for the success of ordinary Portland cement (OPC) is the high availability of the raw materials (limestone and clay) used for its production. However, OPC contributes to a significant amount of anthropogenic CO_2_ emissions, mainly due to the decarbonation occurring during the manufacture of clinker by limestone calcination [1,2]. Moreover, the high clinkerization temperature requires high energy consumption, contributing to the intrinsic energy demand associated with cement production [3]. The need to decrease both carbon footprint and intrinsic energy of the construction industry urges the development of sustainable cements. From a material composition viewpoint, the use of supplementary cementitious materials (SCMs) such as fly ash, silica fume, ground granulated blast-furnace slag, and low-grade clays has shown a proper way to reduce carbon emissions [4,5,6]. However, the limited availability of SCMs conforming to international standards (e.g., EN 197-1) makes it difficult to sustain this strategy widely [7,8]. On the other hand, developing countries are confronted with common challenges to face the high cement demand needed for economic growth [9,10], while the high cost of OPC and the worldwide availability of soil rich in clay minerals in other countries [10,11] necessitate a new generation of cement as a viable alternative to deal with all these challenges. Therefore, recently, the introduction of limestone calcined clay cement (LC^3^) received considerable interest from researchers and decision-makers owing to the reduction of clinker content in this cement [9,12].

LC^3^ is a new type of low-carbon cement that can be made from SCMs available in abundant quantities at low cost, and it can represent an approximate reduction of up to 40% in CO_2_ emissions compared to ordinary Portland cement [10,12,13]. In this context, largely available supplies of clay and limestone facilitate this strategy. The key formulation of LC^3^ consists of the combination of calcined clay and limestone with a mass ratio of 2:1, with the substitution of high amounts of clinker by up to 50 wt %. LC^3^ has shown comparable mechanical properties to those of OPC and the improvement of some durability aspects [7,8,14]. Such cements have not only demonstrated a synergistic effect of calcined clay and limestone, which has led to the reduction of the high amount of clinker consumption, but also lowered energy requirements because the calcination of clays occurs between 700 and 850 °C. Low-grade clays with kaolinite content of up to 40% may be used, and carboaluminate hydrates are generated during hydration that can improve the microstructure, mechanical performance, and durability of concrete [8,12,15,16]. However, calcined clays may increase water demand due to the fineness and large specific surface of the particles, resulting from the sheet-like structure of kaolinite, possibly leading to a loss in the workability of fresh cementitious materials [15,16,17]. Engineering and scientific attempts have been devoted to improving the fluidity of clay-based cement to satisfy the required pumpability of concrete, but the mechanism defining the rheological behavior of clay is still not well understood.

The main difficulty with clay incorporation (i.e., metakaolin) into cementitious material is the water requirement, which is due to its irregular morphological structure [18]. Experimental analyses such as FTIR, DTG and H_2_O mass profiles were used to characterize and investigate structural characteristics of clay minerals [19,20]. The results showed that clays have significant absorption of water. To improve the material workability, several studies were conducted using viscosity-modifying agents in clay-based materials to reduce the yield stress of fresh slurries. Nair et al. [15] pointed out that calcined clays in LC^3^ have increased superplasticizer demand and showed some difficulties in retaining fluidity for extended durations. Basically, fresh cementitious materials exhibit yield stress in which suspensions can flow only if the applied stress is large enough, followed by a gradual microstructural recovery when the stress is removed, with time-dependent recovery (thixotropy) [21,22]. Previous studies have implemented specific experimental methods to determine the variation of important rheological parameters such as static yield stress, elastic modulus, and plastic viscosity with resting time, with the aim of understanding the time-dependent structural recovery of different suspensions [14,16,21,23,24]. Mahaut et al. [21] have measured the evolution of yield stress for cement pastes with time to predict the structuration rate, and they found that the evolution of the structuration at rest is related to the progressive and reversible formation of a solid structure by flocculation. In a clay–water system, it is shown that viscosity mainly depends on the applied shearing and the internal structure of the material due to particle interactions. Moreover, the particle rearrangements during restructuration at rest lead to the formation of a stronger network of interactions, with increasing aging times [25,26,27]. Pignon et al. [28] have demonstrated the existence of a critical shearing, beyond which the clay structure is broken into smaller flocs, while a structural build-up occurs when the system is left to rest. Moreover, the characteristic time of build-up was 3 orders of magnitude less than the breakdown characteristic time. On the other hand, understanding the shrinkage mechanism of fresh pastes is fundamental for predicting deformation after casting. It is noted that clays are more prone to shrinkage when they are mixed with water [29]. A model proposed by Hu et al. [29] showed that the drying shrinkage strain and the shrinkage limit depended on the compressibility of clay particles and the surface tension of the fluid fraction.

Beyond the technical characteristics, the economic advantage of LC^3^ can be relevant compared to OPC due to lower operation cost and WACC (the weighted average cost of capital) [9]. As the recognition of LC^3^’s technical and environmental benefits has grown over the last decade, the level of knowledge of the fresh state properties and the understanding of the interaction between the constituents need to be improved. This paper is devoted to an experimental investigation of the fresh state of limestone calcined clay cement (LC^3^). In order to compare the effect of each component on fresh state behavior, binary systems were characterized in parallel with LC^3^ by a series of experimental procedures, such as rotation and oscillation rheology measurements at different resting times. A similar approach was implemented in two previous studies [30,31]. Different from those studies, we compare the results of rheological measurements with additional tests of the early age behavior of binary and ternary blends by means of Vicat needle measurements of the setting times and by a recently developed method for the assessment of plastic shrinkage. These combined measurements provide an overall view of the early age reactivity and fresh state properties of LC^3^ pastes and of the role of calcined clay and limestone additions in determining the observed behavior.

## 2. Materials and Methods

### 2.1. Materials

**Figure 1:**
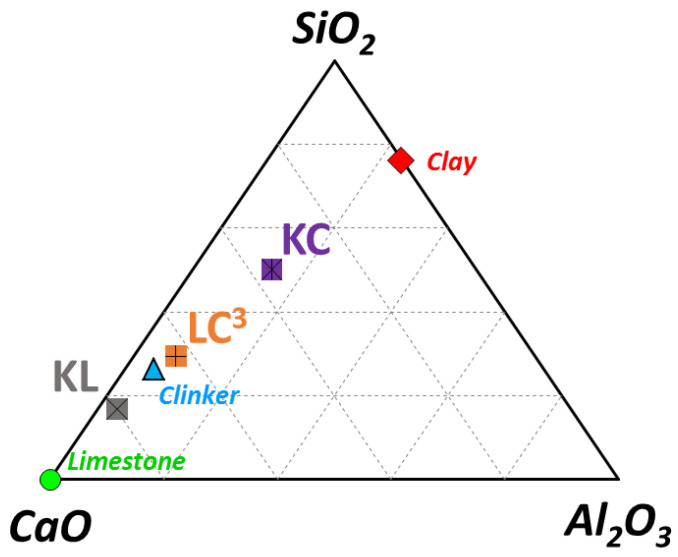
CaO-SiO_2_-Al_2_O_3_ ternary diagram displaying the chemical composition of raw and mixed materials (wt %).

The starting materials used for this study were Portland cement, limestone supplied in the form of waste slurry from marble quarries located in the Apuan Alps in Tuscany (Carrara, Italy) (44°03′08″ N, 10°14′04″ E), and clays obtained from Mûkûrwe’inî- (Nyeri, Kenya) (0.5609° S, 37.0488° E). The chemical compositions of all raw materials are reported in Table 1, and the CaO-SiO_2_-Al_2_O_3_ ternary diagram with respect to the chemical composition (wt %) of raw materials is illustrated in Figure 1.

**Figure 2:**
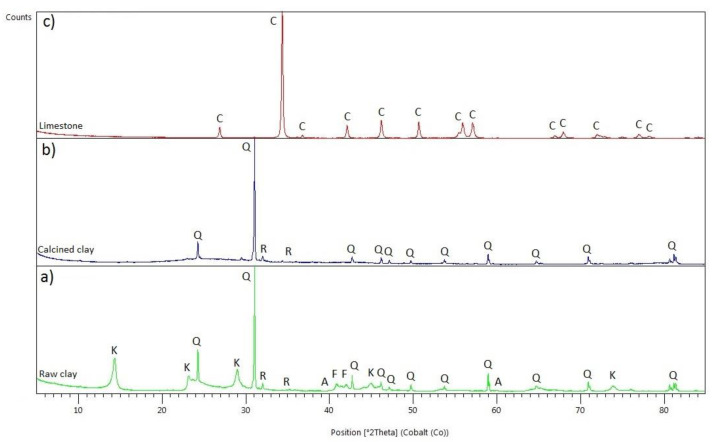
XRD pattern of (**a**) raw and (**b**) calcined clay and (**c**) limestone. Q: quartz; K: kaolinite; R: rutile; F: feldspar; A: anatase; C: calcite.

The mineralogical composition of raw clays has been determined by X-ray diffraction (XRD) using a Panalytical X’Pert Pro diffractometer (Malvern, UK) with Co radiation equipped with an X’Celerator detector, operating within a 2θ range of 5–85°, step size of 0.017°, and a time per step of 100 s. The internal standard method was adopted to quantify the total amount of the amorphous phase in clays using the Rietveld method [32]. The clays were calcined in a laboratory oven for 3 h at 850 °C with a heating rate of 10 °C/min. Figure 2 shows the XRD patterns of the clays and limestone. The kaolinite exhibited a basal distance (*d*_001_) spacing of 7.146 Å that corresponds to a diffraction peak at 14.413° 2θ, as illustrated in Figure 2a. In the calcined clay, the amorphous fraction, metakaolin, is about 40%, whereas quartz is up to 55%.

Four binders were used in this study, in which gypsum was kept constant at 5 wt % while the amount of clinker varied depending on whether the additional cementitious materials were present or not. The total replacement of clinker was kept constant at 45 wt %: the ternary blend (LC^3^) with a ratio of calcined-clay-to-limestone of 2:1 and the binary blends containing 45 wt % of either limestone (KL) or calcined clay (KC) only. The binder compositions, including the reference mixture based on clinker and gypsum (K), are shown in Table 2, and their plotted chemical composition in the CaO-SiO_2_-Al_2_O_3_ ternary diagram is illustrated in Figure 1.

### 2.2. Methods

#### 2.2.1. Setting Time Measurements

The setting time was measured with a Vicat apparatus, as specified by the EAS 148-3:2000 standard [33], in order to investigate the initial reactivity of the blended cements and to select a reasonable range of aging times for the rheological measurements. The four different pastes were cast in the molds specified by the adopted standard and were placed under the Vicat apparatus, equipped with a needle of 1.13 ± 0.05 mm diameter and 300 g mass, provided for the initial setting time determination. The specimens were prepared and tested at a temperature of 25 ± 2 °C and relative humidity higher than 50%. The precision of the measurement depends mainly on the immersion depth of the needle, the measured mass, and the experimental conditions, such as temperature and relative humidity [34]. The needle was lowered gently into the surface of the paste and quickly released and allowed to sink in. The period that elapsed between the time when water was added to the binder and the time at which the needle ceased to penetrate beyond 4 mm from the bottom of the mold was noted as the initial setting time. The final setting time was also determined as the one at which the needle penetrates only 0.5 mm into the surface of the sample.

#### 2.2.2. Rheology Measurements

Four pastes were prepared with a water-to-solid ratio (w/s) of 0.5. The pastes were mixed in an IKA ULTRA TURRAX Tube Drive mixer (Berlin, Germany) for 3 min at 1700 rpm. The rheological tests were started after a total of 5 min from the time of initial contact of the binder with water using a stress-controlled rheometer, Anton Paar MCR 92 (Graz, Austria), equipped with a serrated plate-plate geometry (upper plate of 25 mm diameter and lower plate of 50 mm diameter) at 20 ± 1 °C and a gap of 2 mm between two plates. A solvent trap was used to prevent water evaporation.

The rheological measurements were preceded by preshear and structure recovery intervals to allow for the rejuvenation of the samples and to erase any memory of the shear history exerted by the samples during preparation and loading. The rheological experiments follow two protocols:

(i) Rotational measurements are used to determine yield stress. Shear stress was applied to the sample by presetting the shear rate in a logarithmic ramp. Logarithmic shear rate increasing and decreasing ramps were applied to the sample, from 10^−4^ s^−1^ to 300 s^−1^, with a constant measuring time per point of 5 s. Static yield stress (τ_S_) is evaluated as the stress measured at the lowest shear rate in the increasing shear rate ramp, and dynamic yield stress (τ_D_) is evaluated as the lowest stress measured during the decreasing shear rate ramp. The difference between the two types of yield stress is that τ_S_ reflects the connected structure at rest while τ_D_ measures interactions across the broken interparticle links [30,35]. To quantify the difference between τ_S_ and τ_D_, a thixotropy index (T.I.) can be calculated as T.I. = (τ_S_ − τ_D_)/τ_D_ to evaluate the degree of build-up/breakdown of the structure at different resting times. The thixotropy index (T.I.) is the difference between τ_S_, which measures the cohesion of the system at rest, and τ_D_, which measures the cohesion when the system’s structure is broken down by the applied stress. This microstructural change is, however, a complex phenomenon, whose extent depends on the elapsed time at rest. Therefore, T.I. indicates the degree of the structural build-up after a given aging time.
(ii) Amplitude sweep oscillation measurements are used to determine viscoelastic properties. Constant frequency oscillation with a logarithmically increasing amplitude strain (γ) is applied to the sample. This measurement provides the determination of the extension of the linear viscoelastic region (LVE), where the stress/strain relationship is linear, and the measurement of the storage (G′) and loss (G′′) moduli within this region.
  The preshear, recovery, and measurement protocols are reported in detail in Table 3. The measurements were performed immediately after the recovery step (0 min) and after 30 thirty minutes of aging. One measurement was performed for each formulation only after verifying the reproducibility of the measurements with preliminary tests (the variation was less than 1%). Following the indication obtained from the setting time measurements, τ_S_, τ_D_, and G′ were measured at the sample ages of 0, 30, and 60 min, as reported in Table 3. The resting time of “60 min” refers to the second 30 min step of aging after shear at the first step of “30 min” resting time. During the resting times, the sample builds up the structure that is destroyed during the increasing shear ramps and the large amplitude oscillation of the oscillatory amplitude sweeps. Therefore, the recovery response is quantified by the increase in G′.

#### 2.2.3. Plastic Shrinkage

Plastic shrinkage strain was evaluated on fresh samples (w/s 0.5) placed in cylindrical plastic holders (3 cm height × 1.4 cm diameter) immediately after mixing by means of X-ray imaging (Bruker Skyscan 1172 CT, Billerica, MA, USA). X-ray attenuation images of the samples were acquired for three hours, with 10 min intervals. For each set of samples, the measurements were performed in two modes. The first set of samples was isolated from the surrounding by covering the upper part of the sample holder with a plastic cap, with the aim of preventing moisture loss, called a bleeding regime [36]. The second set of samples was left uncovered, thus allowing evaporation, called a drying regime [36]. In the bleeding regime, plastic shrinkage is mostly associated with gravity-driven particle settling, whereas in the drying regime, the build-up of vertical pressure gradients, triggered by evaporation, contributes to the vertical displacement of the fresh paste [37].

## 3. Results and Discussions

### 3.1. Setting Time

The ESA 148-3:2000 standard [33] prescribes that OPC should have setting times between 100 ± 10 min and 170 ± 10 min, while for blended cements, it should be between 180 ± 10 min and 240 ± 10 min for initial and final setting times, respectively. As it can be seen from Table 4, LC^3^ and KL binders had approximately similar initial setting times. On the other hand, different values were obtained for K and KC (90 and 130 min, respectively). The reference binder K had an early setting time, which is the consequence of faster C-S-H nucleation, leading to a build-up of bridges between cement particles [35,38]. Replacing equivalent clinker fractions with calcined clays or limestone delays the initial and final settings. However, the setting modifications are different for the two. As for most kaolinitic materials that have the ability to absorb water and poor pozzolanic reactivity at an early age [18,39,40], the usage of calcined clays can modify the chemical equilibria and delay the hydration rate due to the lower fraction of hydrating clinker, thus delaying portlandite precipitation and the subsequent setting [40,41]. In the case of KL, a limestone addition reduced the initial setting time by 20 min compared to calcined clays (KCs). Limestone powder can accelerate the hydration of clinker, notably C_3_S, by providing nucleation sites for the precipitation of calcium–silicate hydrates [42]. On the other hand, the high affinity of clinker (C_3_A) and limestone (CO_2_) can modify the process of hydration and form carboaluminate phases rather than sulphoaluminates [43,44]. As more gypsum (SO_3_) is left in the solution due to the formation of carboaluminate instead of ettringite, the rate of C_3_A dissolution is decreased, and the retardation of hydration reactions and setting is achieved [45,46]. In previous studies, it has been reported that the partial replacement of OPC by the limestone and metakaolinite (Al_2_O_3_.SiO_2_), present in calcined clays, may induce further hydration reaction [9,39]. The presence of limestone in the binder causes a significant reactivity with the calcium aluminates present in both the calcined clays and clinker and with portlandite to form aluminate hydrate phases during the early stage [39,47]. The synergy of the blend components appeared to have a significant effect on the fresh state behavior of LC^3^. However, other aspects such as substitution fraction, filler effect, and the metakaolin-to-portlandite ratio can equally contribute [16,39,47].

The Vicat test cannot give an exact indication of the rheological properties such as yield stress and viscosity and their evolution with time, but it can help to better understand the mechanism of particle interactions within the sample. Some authors [48] have noted that the initial setting time correlates with the elastic modulus (G′) of the material. Recent studies [38,48,49,50] have shown that G′ increases significantly with the beginning of setting due to C-S-H and portlandite nucleation. However, this mechanism does not necessarily coincide with the increase in yield stress before setting [48,50,51]. These findings suggest different mechanisms influencing the rheological properties of the system: (i) the structure of fresh cement can be controlled mainly by the nucleation and growth of C-S-H, (ii) absorbed and entrapped water in the particle agglomerates (flocs) formed by the flocculation of clays causes an increase in yield stress and elastic modulus, and (iii) the use of limestone can reduce the yield stress and elastic modulus due to the dilution effect [30,48,52,53].

Nevertheless, the rheological properties identified by the Vicat test are not sufficient to describe the rheological behavior of fresh slurries due to the difference of forces applied to the material throughout the measurement. The rheometer is able to detect the changes of structural organization inaccessible to the Vicat needle [52,54].

### 3.2. Rheological Properties

#### 3.2.1. Yield Stress

**Figure 3:**
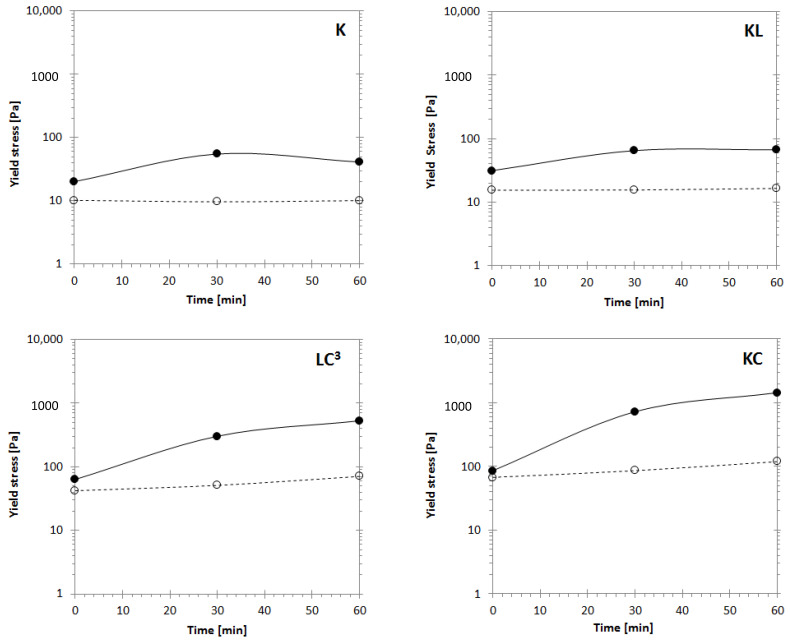
Effect of resting time on static yield stress τ_S_ (filled circles) and dynamic yield stress τ_D_ (open circles) determined from the flow curves.

**Figure 4:**
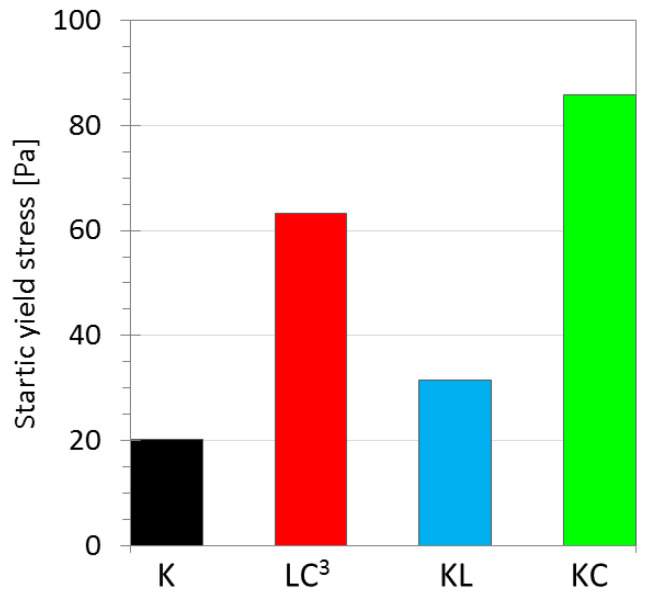
Static yield stress τ_S_ of sheared binders at an early age (0 min).

The influence of clinker replacement by limestone and calcined clay on yield stress evolution with time is illustrated in Figure 3. At an early age (0 min), τ_S_ is higher for binders, including calcined clay, LC^3^, and KC, respectively, displaying values three to four times larger than the one of K (Figure 4). It can be seen from Figure 3 that τ_S_ increases with different rates for the different binders during resting time. After 30 min of rest, τ_S_ of the binder K reached a value of 55.1 Pa (two times higher than at 0 min) and slightly decreased after 60 min of resting time in a steady range of values. This reduction of τ_S_ can be explained by flocculation and bridging. After mixing (0 min), the system exhibited a low τ_S_ value. Then, it started to flocculate during the first minutes of rest, and hydrated products (i.e., ettringite and C-S-H) started to nucleate and bind cement particles together, thus increasing τ_S_ after 30 min of rest. By shearing the system, such formed bridges are broken [55]. During the following 30 min of rest, the system started to rebuild its structure in the same manner. However, most of the sulfates were consumed during the first minutes [56,57] and, therefore, only C-S-H nucleates can bind the cement particles. This nucleation happens before the acceleration of hydration when fresh paste K starts to set at 90 min, as shown in Table 4. As a result, a lower τ_S_ was obtained at 60 min. Similar observations were reported elsewhere [58,59,60]. The same trend was observed for the binder with limestone only (KL). However, the use of calcined clay increased, to a larger extent, the τ_S_ of binders KC and LC^3^ after 30 min and continued to increase its value up to 60 min, as can be observed in Figure 3. The τ_D_ of each sample remained almost equal at the different resting times, confirming that the build-up/breakdown of the structure is reversible when sufficient shearing is applied. Higher τ_D_ values were recorded when calcined clay was used.

The different rates of τ_S_ increase during the resting time can reveal the difference in the build-up/breakdown mechanism. The presence of calcined clay in binders (LC^3^ and KC) leads to a high rate of τ_S_ increase with time, which suggests a high structuration rate of the material at rest. The combination of limestone with calcined clay decreases the τ_S_ of binder LC^3^ compared to KC due to the presence of limestone. The individual effect of each material, calcined clay and limestone, on yield stress can be clearly seen in Figure 3. In general, the binary limestone blend (KL) showed a low difference between τ_S_ and τ_D_ at different resting times compared to the binary calcined clay blend (KC).

The thixotropy index (T.I.) values at an early age (0 min), from Table 5, suggested that the binder with calcined clay (KC) had lower thixotropy, and limestone increased the thixotropy of KL compared to that of reference K. The use of calcined clays had no significant effect on thixotropy at an early age, which suggests that flocculation is the sole mechanism controlling the structure build-up of the KC system and the shear effectively destroys the initial structuration. In contrast, binder KL showed the highest thixotropy, larger than reference K, which could be due to the chemical reactions occurring in the presence of limestone. Some authors [61] have documented that the incorporation of limestone powder displayed an increase in the rate of cement hydration, mainly with respect to C_3_S and C_3_A, at an early stage. However, the chemical effect of limestone remained minor since the aluminate phase was limited in the used cement, although the addition of SCMs increased Al concentration. This effect can be clearly seen during the longer resting time, as discussed later.

The T.I. values revealed that all binders had an increase in thixotropy after the system was left at rest. This indicates that the build-up and restructuration between the particles have occurred during rest. However, binder K showed lower T.I. at 60 min than at 30 min. This can be due to the structural breakdown associated with the bridge’s breakage. The structural build-up rate correlated to the hydrated products (i.e., C-S-H) was much slower than the thixotropic build-up (i.e., Van der Waals attraction, electrostatic repulsion, and steric hindrance) [55].

From 30 to 60 min, LC^3^ and KC binders showed a larger increase in T.I. The particles in calcined-clay-based systems had the time to rearrange and achieve stronger interaction forces, which are reflected in the increase of τ_S_ and thixotropy from 30 to 60 min rest. Binder KC showed a thixotropy approximately four times higher than that of binder K after 60 min resting time. Therefore, the mechanism underlying the fresh state response of cement paste with calcined clays could be associated with the increase in yield stress due to flocculation, as confirmed by several studies [62,63]. On the contrary, limestone showed the lowest thixotropy at 30 min of rest and kept constant after 60 min resting time. The difference in the rheological properties of limestone and calcined clay can manifest when the two materials are combined. Limestone can reduce the yield stress and thixotropy of the LC^3^ system. Similar conclusions were found in other studies [30,64].

#### 3.2.2. Viscoelastic Properties

**Figure 5:**
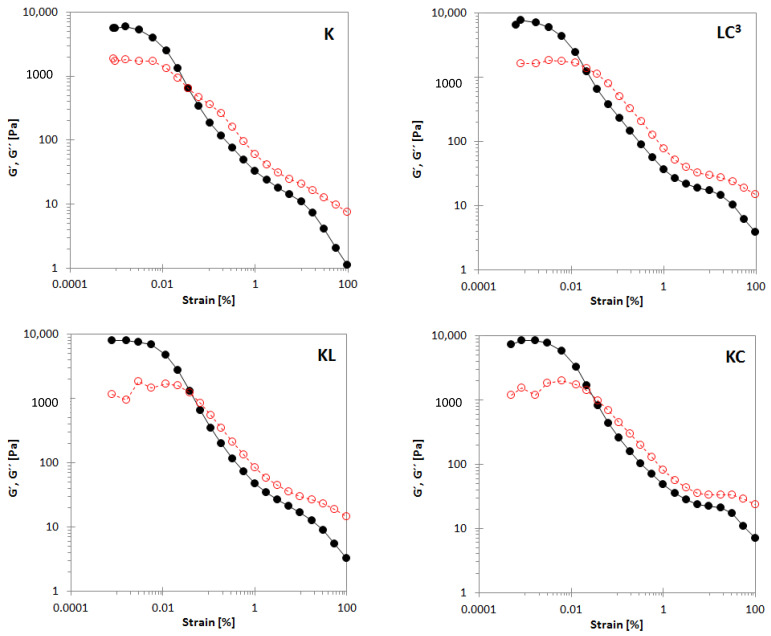
Elastic modulus G′ (filled circles) and viscous modulus G′′ (open circles) vs. oscillation strain amplitude γ for all samples at an early age (0 min).

Oscillatory strain sweep at an early age: the variation of storage (G′) and loss (G″) moduli with strain for all binders at 0 min are shown in Figure 5. It can be seen that all binders displayed an LVE response and high elasticity (G′ > G″) at a small amplitude of deformation, reflecting a solid-like state or a structured network of the material. The limit of LVE response is characterized by a critical strain (γ_cr_) from where G′ and G″ decrease and the material begins to yield. At larger strain, G′ and G″ continue to decrease until crossover, when G′ = G″ and the material becomes liquid-like, reflecting a complete microstructure collapse. It is clear that all binders started to yield in the same range of γ_cr_—K: 0.0031%, LC^3^: 0.0033%, KL: 0.0056%, and KC: 0.0030%. However, the corresponding G′ revealed an increase with a monotonic order of K: 5272.4 Pa, LC^3^: 5945.5 Pa, KL: 6892.5 Pa, and KC: 7850.0 Pa. The values of γ_cr_ and G′ depend on the interparticle interactions and the network structure [1,65,66]. Calcined clay has significantly increased the interparticle structure and led to a strong network due to flocculation and high water adsorption [30,67], whereas its combination with limestone led to a decrease in network formation.

**Figure 6:**
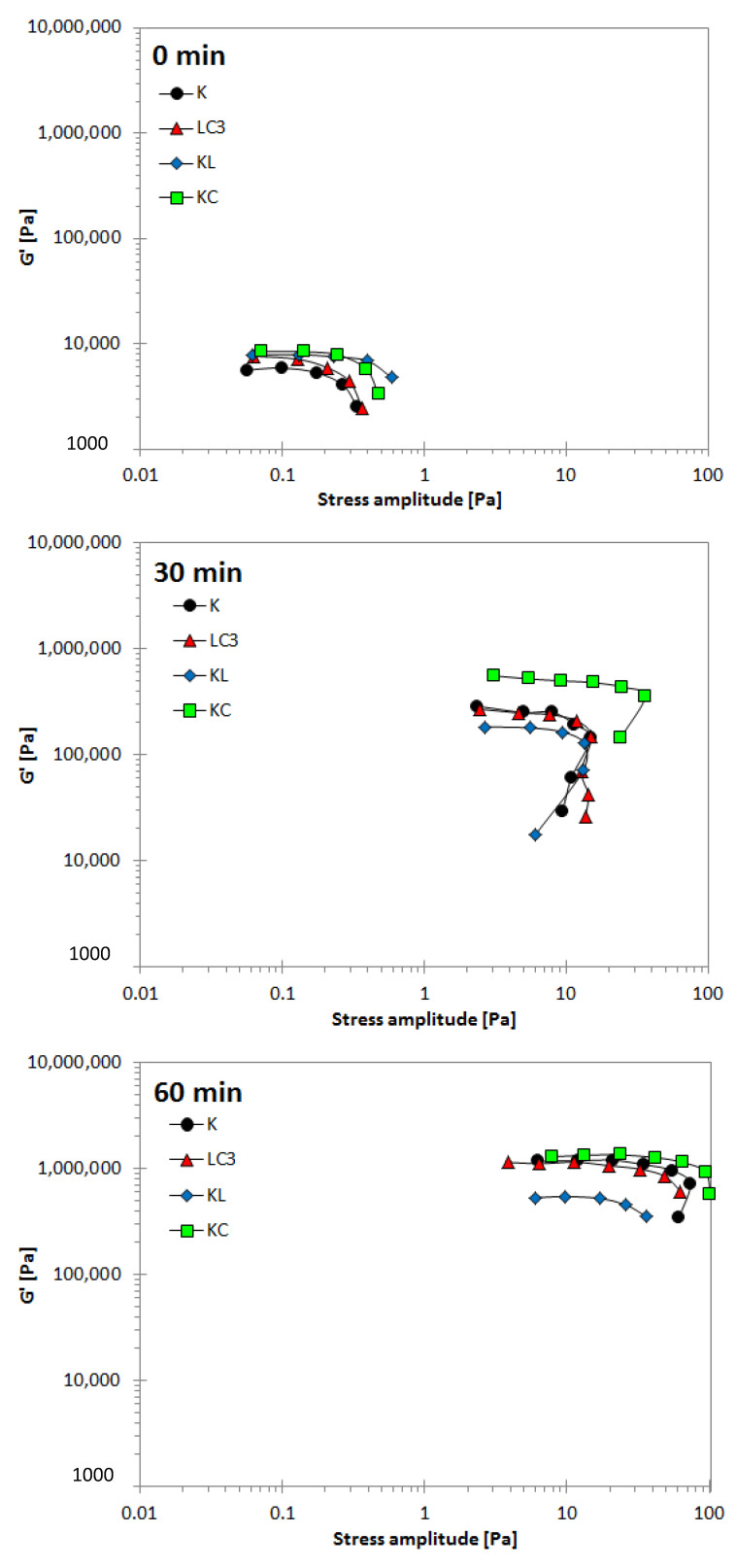
Elastic modulus vs. stress amplitude needed to yield the binders at different resting times.

Oscillatory stress amplitude-effect of resting time; the measured elastic modulus, as a function of stress amplitude at different resting times, is presented in Figure 6. At an early age (0 min), the LVE region of all binders ended at a stress amplitude of about 0.01–0.4 Pa, with G′ lower than 10^4^ Pa. Upon increasing the resting time, the limit of LVE regions were observed at stress amplitude of about 7.9–35.8 and 16.9–54.9 Pa after 30 and 60 min, respectively, with an increment of G′ in a range of 10^5^–10^6^ Pa for the different resting times. A high restructuration rate can be evaluated through the increment of G′ and τ_cr_ (critical shear stress amplitude, where G′ starts to drop; static yield stress obtained from the oscillation regime) values at different resting times, as summarized in Table 6. At 30 min of rest, calcined clay (KC) exhibited a highly structured network (G′ = 3.55 × 10^5^ Pa and τ_cr_ = 35.74 Pa). In contrast, limestone (KL) showed lower values of G′ = 1.63 10^5^ Pa and τ_cr_ = 9.41 Pa, thus a weaker structure, while the combination of the two materials balanced the structure build-up of LC^3^ (G′ = 2.08 × 10^5^ Pa, τ_cr_ = 11.83 Pa). The highest value of τ_cr_ (54.83 Pa) was obtained for binder K at a later age. A possible explanation is the one stated in a previous study [49], where it was observed that the colloidal interactions between the cement particles dominate at an early age, while the rigidification of C-S-H bridges may become the dominant factor at later stages. This finding can be confirmed by the early initial setting time of binder K.

The G′ values of all samples were increased from 30 to 60 min as follows: K = 2.52 × 10^5^ to 9.50 × 10^5^ Pa, LC^3^ = 2.08 × 10^5^ to 9.87 × 10^5^ Pa, KL = 1.63 × 10^5^ to 5.21 × 10^5^ Pa, and KC = 3.55 × 10^5^ to 1.27 × 10^6^ Pa. It is noted that the binders K and LC^3^ exhibit comparable values of G′, while KL has lower values and KC has higher values. This difference can be explained by three different mechanisms: (i) chemical reactions associated with the combination of limestone and SCMs rich in aluminates, such as calcined clay [61], (ii) a dilution effect in the presence of a high limestone fraction, resulting in a larger amount of free water in the system [61], and (iii) an increase of elastic modulus due to the increase of flocculation strength and water adsorption in the presence of a high fraction of calcined clays [62,63].

**Figure 7:**
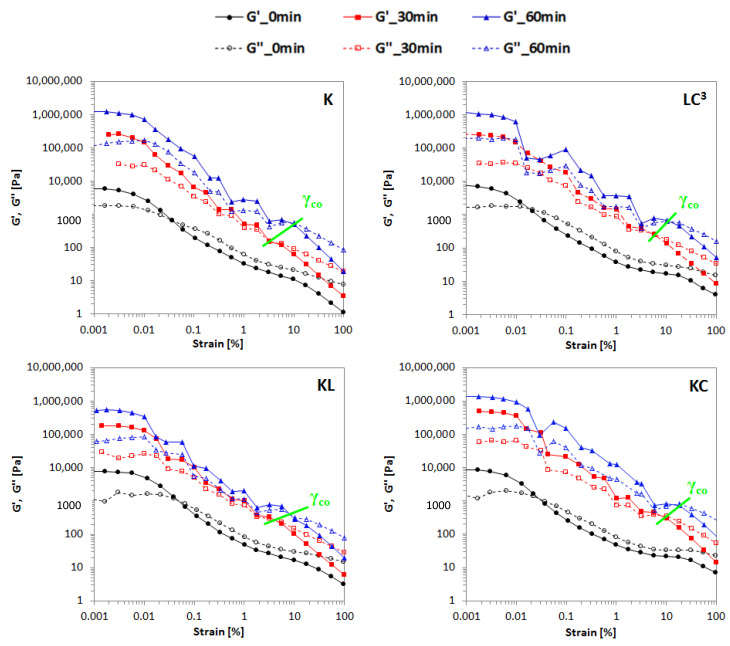
Storage (G′, solid line) and loss (G′′, dashed line) moduli vs. oscillation strain for binders at different resting times. γ_co_ is the crossover point, when G′ = G″.

Oscillatory strain amplitude-effect of resting time; the evolution of G′ and G″ moduli of various binders with strain amplitude and resting time is illustrated in Figure 7. G’ and γ_cr_ of all binders increased with the continuous resting time, as showed in Table 6. In Figure 7, it can be seen that the amplitude sweep curves showed some abnormal points that departed from the usual trend when strain was increased from γ_cr_ (~0.01%) to γ_co_ (crossover point when G′ and G″ are equal, ~10%) after an age of 30 and 60 min. This trend, which is different from that observed with samples at an early age (0 min), indicated that a sample failure occurred during deformation and the structure became fragile. However, the fragile sample built up its structure and became stronger with time at rest, leading to a progressive increase of γ_cr_ and also γ_co_, where nearest neighbor particle links start to be broken, eventually leading to flow.

The use of calcined clay in the binary system resulted in the development of higher particle reorganization during restructuration at rest, thus leading to the formation of a stronger network. On the other hand, the use of limestone alone resulted in lower rearrangement and restructuration of particles at rest, leading to lower network strength after the breakdown process compared to the other systems. The combination of two materials, calcined clay and limestone, therefore, has balanced the breakdown/build-up process due to the progressive and reversible formation of network interactions.

### 3.3. Plastic Shrinkage

**Figure 8:**
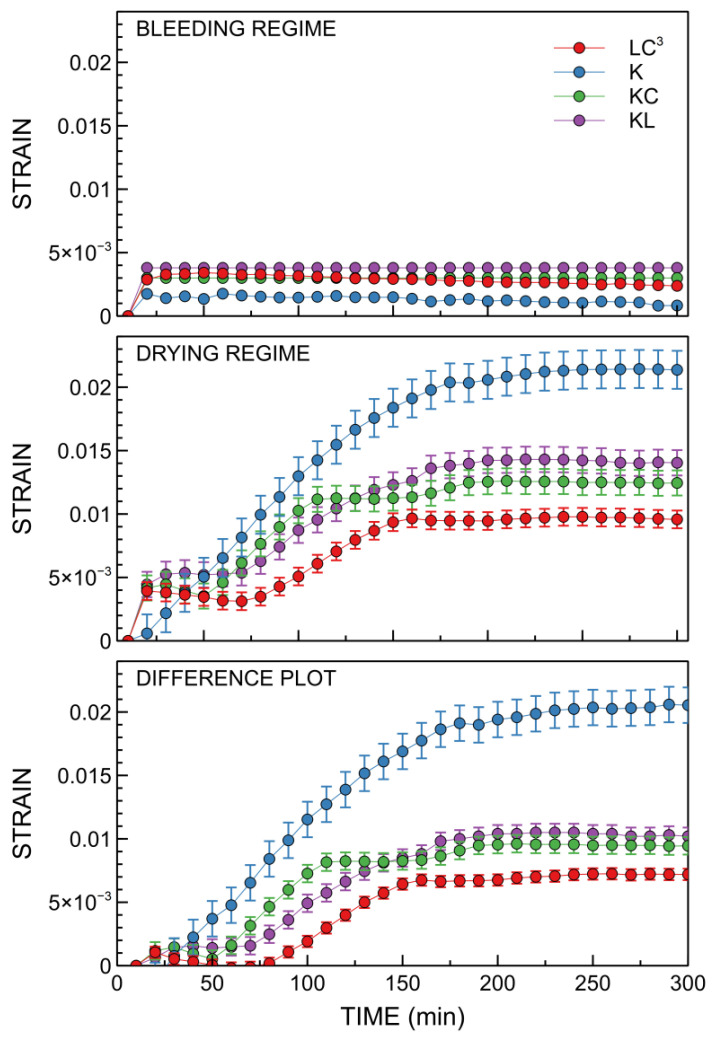
Plastic shrinkage measured by X-ray imaging for the fresh binders. Error bars are based on the estimated precision [37].

Results of the plastic shrinkage measurements are displayed in Figure 8. It can be observed that the contribution to the strain measured in the bleeding regime, associated with particle settling, increases during the first time scale in all systems in which Portland clinker is partially replaced and reaches a steady-state at about 20 min, which could be attributed to enhanced particle flocculation and settlement. In the drying regime, the opposite behavior is observed, with binder K displaying a significantly higher strain. The LC^3^ system outperforms the K system in terms of early shrinkage values, with a reduction of strain measured at three hours by more than a factor of 2.

In the bottom part of Figure 8, the difference between strains measured in the two configurations is reported. By subtracting the contribution associated with particle settling, the sole contribution of capillary stresses arising due to evaporation can be observed in this plot. By comparison with the upper part of Figure 8, it can be concluded that most of the observed shrinkage is related to such capillary pressure. Strain increases immediately, with a steep gradient for Portland clinker, whereas such a steep increase is delayed in the other binders.

The plastic shrinkage process is exemplified in the mechanism proposed in a previous study [68]. After casting, the particles settle due to gravity, causing the formation of a bleed layer, which subsequently starts to evaporate (*initial drying period*). A longer period was observed for binders with SCMs (above 50 min) due to a high bleeding rate. When the evaporation rate is higher than the bleeding rate, menisci start to form at the surface and induce a build-up of capillary pressure, leading to the initiation of shrinkage [36] (time-lapse animations are available in the Supplementary Materials). At this stage, water is transported from within the pore structure to the surface and continues to evaporate (*constant rate period*). This period occurred immediately or over a very short time scale after casting binder K (without SCMs), displaying significant shrinkage due to rapid capillary pressure development. However, the presence of SCMs tends to reduce the plastic shrinkage of binary and ternary systems. This suggests that the reduction of early age shrinkage, observed for the blended cements with SCMs, might be associated with pore network refinement [13,69] that delays the formation and subsequent evaporation of the bleed layer. Consequently, proportional compaction of the solid network, which is developed upon growing capillary pressure under evaporation, is expected. Notably, in soil–fluid systems, it has been demonstrated that the shrinkage is driven by the evaporation rate, and the shrinkage limit is controlled by solid network compressibility [29]. Therefore, binders with SCMs tend to have a finer pore structure than ordinary Portland binder K, which can result in higher resistance to capillary pressure and, hence, lower shrinkage [70,71]. The menisci move into the pore structure (*critical point*) due to the ongoing evaporation, resulting in a reduced interparticle distance. After this point, the capillary pressure is no longer able to compact the solid network and the settlement becomes negligible; thus, there is no more vertical shrinkage (*falling rate period*).

Concerning the relationship between plastic shrinkage and setting, the fraction of strain associated with particle settling occurs within the first 10 min, well before the initial setting. For capillary-stress-induced shrinkage, the initial setting time for all binders occurs within the evolution of capillary pressure due to the ongoing evaporation, while a plateau is approached for binder K near its corresponding final setting time (174 min), but no clear correlation is observed for other binders, for which the plateau is reached some time in between initial and final setting times.

## 4. Conclusions

This paper presents an overview of the behavior of SCMs from the casting to the setting of binary and ternary systems. Blended cements (w/s 0.5) were evaluated by Vicat needle, rheometer, and X-ray imaging to determine setting times, rheological properties, and the evolution of early shrinkage for the fresh pastes.

Binders with SCMs had a higher initial setting time due to the low fraction of clinker. The use of a high calcined clay fraction in binary systems induced an overall delay in setting, while the equivalent replacement of the clinker fraction by limestone or limestone/calcined clay caused a smaller setting time delay compared to the reference binder. The difference of setting in the presence of SCMs can be explained by chemical equilibria, progressive flocculation, and water demand. While the presence of calcined clay increases particle flocculation, limestone addition has the benefit of enhancing the fluidity of the fresh pastes. The rheological measurements have quantified the structural changes of sheared systems after rest (aging). Before the initial setting time, the clinker paste without SCMs exposed an unexpected decrease in static yield stress and the thixotropy index. This is attributed to the bridge’s breakage (i.e., C-S-H) between particles by shearing. Therefore, the structural build-up rate correlated to the hydrated products is much slower relative to the thixotropic build-up (i.e., Van der Waals attraction, electrostatic repulsion, and steric hindrance). The development of particle reorganization during restructuration was significantly increased by using a high calcined clay fraction, which leads to the formation of a strong network, whereas limestone provided a low rearrangement of the particle network, leading to lower structure strength. The combination of two SCMs, consequently, has balanced the breakdown/build-up process of the structure due to the progressive and reversible formation of network interactions. The early shrinkage of fresh pastes is significantly influenced by growing capillary pressure under evaporation. During drying, the surface tension of the fluid fraction caused by evaporation contributes to the increase of plastic shrinkage, while its limit is controlled by pore structure refinement and compaction yielded by SCMs.

This contribution provides not only a quantitative and qualitative assessment of combined limestone and calcined clay as a high fraction (up to 50 wt %) replacement of OPC but also delivers an overview of the fresh state properties of LC^3^ from casting to setting. Therefore, more research and testing on the production, environmental sustainability, and cost-effectiveness of LC^3^ can lead to an effective solution for the global cement market. 

## Figures and Tables

**Table 1:** Chemical composition of raw materials (wt %).

Material	SiO_2_	TiO_2_	Al_2_O_3_	Fe_2_O_3_	MnO	MgO	CaO	Na_2_O	K_2_O	P_2_O_5_	L.O.I
Clay	69.07	0.52	21.43	1.85	0.11	0.13	0.20	0.15	0.44	0.07	6.00
Clinker	23.67	0.35	4.69	4.06	0.10	2.07	61.43	0.69	1.53	0.00	1.41
Limestone	0.00	0.00	0.10	0.00	0.00	0.60	55.90	0.00	0.01	0.00	43.4

**Table 2:** Summary of the proportion of various binders (wt %).

Binder	Clinker	Limestone	Calcined Clay	Gypsum
K	95	-	-	5
LC^3^	50	30	15	5
KL	50	45	-	5
KC	50	-	45	5

**Table 3:** Summary of the testing protocols.

Protocol Test	Rheological Properties	Action	Resting Time
Rotational regime	Static yield stress, Dynamic yield stress	Pre-shearing: Shear rate: 100 s^−1^, (30 s) Recovery: Strain: 0.0001%, Frequency: 1 Hz, (120 s) Shear rate (logarithmic ramps): Ramp up: 0.0001–300 s^−1^, (60 s) Ramp down: 300–0.0001 s^−1^, (60 s)	0 min (after recovery), 30 min, 60 min
Oscillation regime	LVE region, Elastic modulus	Pre-shearing: Strain: 10%, Frequency: 1.5 Hz, (30 s) Recovery: Strain: 0.0001%, Frequency: 1 Hz, (120 s) Shear strain: Logarithmic ramp: 0.0001–100%, 1 Hz, (time set by device)	0 min (after recovery), 30 min, 60 min

**Table 4:** Initial and final setting times (in min) of blended cements.

Binders	Initial Setting Time	Final Setting Time
K	90	174
LC^3^	114	234
KL	109	224
KC	130	280

**Table 5:** T.I. of binders at different resting times.

Resting Time	K	LC^3^	KL	KC
0 min	0.97	0.49	1.02	0.27
30 min	4.70	4.91	3.16	7.43
60 min	3.06	6.47	3.04	11.10

**Table 6:** Summary of the rheological properties obtained from the oscillation regime for binders at different resting times.

Binders	Rheological Properties	0 min	Resting Time 30 min	60 min
K	τ_cr_ (Pa)	0.17	7.91	54.83
	γ_cr_ (%)	3.10 × 10^−3^	3.11 × 10^−3^	5.63 × 10^−3^
	G′ (Pa)	5.27 × 10^3^	2.52 × 10^5^	9.50 × 10^5^
	γ_co_ (Pa)	3.64 × 10^−2^	3.20	10.20
LC^3^	τ_cr_ (Pa)	0.12	11.83	32.61
	γ_cr_ (%)	3.30 × 10^−3^	5.60 × 10^−3^	9.82 × 10^−3^
	G′ (Pa)	5.94 × 10^3^	2.08 × 10^5^	9.87 × 10^5^
	γ_co_ (Pa)	2.14 × 10^−2^	5.78	10.50
KL	τ_cr_ (Pa)	0.39	9.41	16.96
	γ_cr_ (%)	5.60 × 10^−3^	5.71 × 10^−3^	9.98 × 10^−3^
	G′ (Pa)	6.89 × 10^3^	1.63 × 10^5^	5.62 × 10^5^
	γ_co_ (Pa)	3.95 × 10^−2^	3.20	7.50
KC	τ_cr_ (Pa)	0.24	35.74	42.15
	γ_cr_ (%)	3.00 × 10^−3^	9.89 × 10^−3^	1.00 × 10^−2^
	G′ (Pa)	7.85 × 10^3^	3.55 × 10^5^	1.27 × 10^6^
	γ_co_ (%)	2.21 × 10^−2^	10.30	18.00

## Data Availability

The data presented in this study are available on request from the corresponding author.

## Supplementary Materials

The following supplementary materials are available online at http://researchdata.cab.unipd.it/id/eprint/470.
